# Pulmonary EV miRNA profiles identify disease and distinct inflammatory endotypes in COPD

**DOI:** 10.3389/fmed.2022.1039702

**Published:** 2022-12-15

**Authors:** Hannah Burke, Doriana Cellura, Anna Freeman, Alex Hicks, Kris Ostridge, Alastair Watson, Nicholas P. Williams, C. Mirella Spalluto, Karl J. Staples, Tom M. A. Wilkinson

**Affiliations:** ^1^Faculty of Medicine, University of Southampton, Southampton, United Kingdom; ^2^NIHR Southampton Biomedical Research Centre, University Hospital Southampton, Southampton, United Kingdom; ^3^Translational Science and Experimental Medicine, Research and Early Development, Respiratory and Immunology, BioPharmaceuticals R&D, AstraZeneca, Gothenburg, Sweden

**Keywords:** COPD, extracellular vesicles, microRNA, inflammatory endotypes, early diagnostics

## Abstract

**Introduction:**

Chronic obstructive pulmonary disease (COPD) is a heterogeneous condition without effective disease modifying therapies. Identification of novel inflammatory endotype markers such as extracellular vesicles (EVs), which are important intercellular messengers carrying microRNA (miRNA), may enable earlier diagnosis and disease stratification for a targeted treatment approach. Our aim was to identify differentially expressed EV miRNA in the lungs of COPD patients compared with healthy ex-smokers and determine whether they can help define inflammatory COPD endotypes.

**Methods:**

EV miRNA were isolated and sequenced from ex-smoking COPD patients and healthy ex-smoker bronchoalveolar lavage fluid. Results were validated with RT-qPCR and compared to differential inflammatory cell counts.

**Results:**

Expression analysis identified five upregulated miRNA in COPD (miR-223-3p, miR-2110, miR-182-5p, miR-200b-5p and miR-625-3p) and three downregulated miRNA (miR-138-5p, miR-338-3p and miR-204-5p), all with a log2 fold change of >1/−1, FDR < 0.05. These miRNAs correlated with disease defining characteristics such as FEF 25–75% (a small airways disease measure) and DLCO% (a surrogate measure of emphysema). Receiver operator curve analysis demonstrated miR-2110, miR-223-3p, and miR-182-5p showed excellent combinatory predictive ability (AUC 0.91, *p* < 0.0001) in differentiating between health and mild COPD. Furthermore, miR-223-3p and miR-338-3p correlated with airway eosinophilia and were able to distinguish “pure eosinophilic” COPD from other airway inflammatory subtypes (AUC 0.94 and 0.85, respectively).

**Discussion:**

This is the first study to identify differentially expressed miRNA in COPD bronchoalveolar lavage fluid EVs. These findings suggest specific lung derived EV miRNA are a strong predictor of disease presence even in mild COPD. Furthermore, specific miRNA correlated with inflammatory cell numbers in COPD, and may have a role in defining inflammatory endotypes for future treatment stratification.

## Introduction

Chronic obstructive pulmonary disease (COPD) affects 384 million people worldwide and is the third leading cause of death globally (1). The burden of COPD is predicted to increase over the next few decades due to continued exposure to COPD risk factors, such as tobacco smoke and an aging population (2). There is growing interest in early COPD as it is envisaged that preventative efforts and treatment can modify its clinical course. Furthermore, it is recognised that our current spirometric diagnostic classifier of forced expiratory volume in 1 s (FEV1)/forced vital capacity (FVC) ratio (3) is a crude tool, which may miss early disease and correlates poorly with symptoms particularly in mild disease (4). Therefore exploring additional diagnostic strategies may improve early diagnosis and provide potential insights into the underlying biology of the disease (5).

Endotypes describe a distinct pathophysiological mechanism at a cellular and molecular level, leading to a clinical phenotype of disease. Despite the clinical heterogeneity of COPD, it has proved difficult to identify distinct endotypes of disease which relate to outcome, however, different inflammatory patterns have been described in COPD and are referred to as “*inflammatory endotypes*” (6). Most patients with COPD have increased numbers of neutrophils and macrophages in their lungs reflecting the inflammatory nature of the disease (5, 6). Some patients also have elevated numbers of eosinophils, which are associated with more frequent exacerbations (7–9) and importantly predict good response to corticosteroid treatment (7, 10). However, studies have failed to show the same improvement with other treatments targeting eosinophilia [e.g., anti-interleukin (IL)-5 monoclonal antibodies] (11). Therefore, further research is needed to understand the mechanisms behind these inflammatory endotypes in COPD.

Extracellular vesicles (EVs) are key intercellular messengers and have been identified as playing an important role in inflammatory regulation within the lungs (12, 13). EVs have been identified as novel disease biomarkers due to their capacity to reflect the parent cell’s physiological state and microenvironment, as well as being highly stable in bodily fluids [such as bronchoalveolar lavage fluid (BALF) (14)], with the ability to package an array of disease associated molecules, such as microRNA (miRNA) (15, 16). MiRNAs are small non-coding RNA molecules that control post-transcriptional gene expression (17). MiRNAs have emerged as potent modulators of inflammation (12) and their utility as biomarkers in COPD is being explored (18, 19). Furthermore, studies have already demonstrated the use of circulating EVs (e.g., microvesicles) as possible biomarker candidates in COPD (20, 21). However, to date, no study has directly sampled and characterised EV miRNA from the lungs of patients with COPD. Exploring the predictive ability of these miRNA to distinguish health from disease in mild COPD and evaluating their relationship with inflammatory endotypes may lead to new insights into inflammatory disease mechanisms and identify new innovative targets for therapy.

## Materials and methods

### Study cohort

**FIGURE 1:**
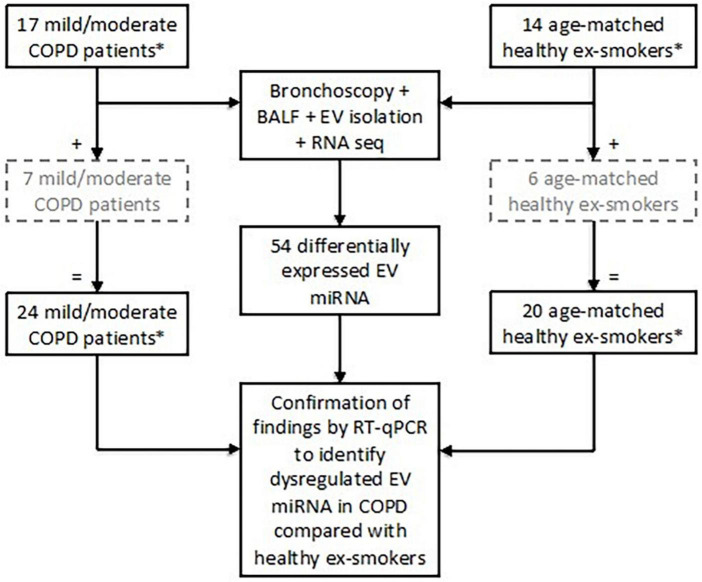
Subject enrolment and tests performed in the study to assess extracellular vesicle (EV) microRNA (miRNA) expression. Subjects included in the grey, hashed boxes were recruited later and therefore only underwent testing for EV miRNA via RT-qPCR. *All subjects were ex-smokers having given up smoking at least 6 months prior to study enrolment and with at least a 10-pack year smoking history.

Twenty-four subjects with stable mild or moderate COPD as defined by the GOLD Guidelines (3) were recruited. All subjects were ex-smokers (>10-pack year history), having given up smoking at least 6 months prior to study enrolment (5, 22, 23). Post-bronchodilator spirometry was used to assess airflow obstruction with an FEV1/FVC ratio < 0.7 and an FEV1 of ≥50% predicted value required for enrolment as COPD subjects (3). Exclusion criteria included a history of other pulmonary disease (e.g., asthma), α-1-antitrypsin deficiency, use of long-term antibiotics/oral corticosteroids, or an exacerbation within a month prior to study enrolment. Twenty healthy, age-matched ex-smokers (>10-pack year history) were recruited as a control group (Figure 1).

### Sampling

Bronchoscopy sampling was performed on an outpatient basis and was approved by and performed in accordance with National Research Ethics Service South Central ethical standards–Hampshire A and Oxford C Committees (LREC no: 15/SC/0528) (24). Bronchoalveolar lavage was performed by instilling 100 mL of pre-warmed 0.9% sodium chloride into a pre-specified lobe (identified on high-resolution computer tomography as the lobe with the most evidence of small airways disease) in 20 ml aliquots and recovered by gentle aspiration. A total of 15 ml BALF per subject was processed for EV analysis, where differential cell counts were undertaken and EVs were isolated using a combination ultrafiltration and size exclusion chromatography. EVs from a sub-group of subjects (*n* = 9; COPD, *n* = 3, where excess BALF available) were characterised by CD9 ELISA, TEM and Western blot for the presence of CD63 and absence of the endoplasmic reticulum marker calnexin (Supplementary Figure 1).

### RNA isolation

Total RNA from BALF EVs was extracted using miRNeasy^®^ Serum/Plasma Advanced kit (Qiagen^®^, Manchester, UK) according to the manufacturer’s instructions. Qiaseq miRNA Library Quality control Spike-ins solution (Qiagen^®^) were added to each of the EV samples prior to isolation to assess the quality of RNA isolation across samples by qPCR. Pearson correlation analysis of the 52 RNA spike in Cq values demonstrated excellent correlation, with *R*^2^ values between 0.94 and 1.0 for all samples.

### miRNA sequencing

Small RNA sequencing library preparation was performed using Qiaseq™ miRNA Library Kit (Qiagen^®^) on EV RNA samples from 17 patients with COPD and 14 healthy ex-smokers (as not all EV miRNA samples were available at the time of sequencing). The quantity and quality of miRNA libraries were determined using a high sensitivity DNA chip on an Agilent^®^ Bioanalyser 2100. Sequencing was performed on the NextSeq500 instrument (Illumina^®^, Chesterford, UK). Average reads per sample were 2.8 million. Quality control of raw RNA sequencing data was performed using FastQC (v0.11.7) and quality control of raw reads of adapter sequences was done using cutadapt (v1.11). Alignment was performed using bowtie2 (v2.2.2) to the human reference genome (GRCh37/hg19) and miRNA to miRbase (v_20) with an average genome-mapping rate 53.4%. During library preparation and sequencing, the samples were evenly distributed over the batches based on disease/control status.

### Statistical analysis of small RNA sequencing data

The small RNA sequencing analysis was performed with R (v3.8.2). Prior to filtering, 2308 miRNA were detected across all samples. All filtering was performed on log-transformed counts per million (CPM) data, with lowly expressed miRNAs were filtered out, using a cut-off of >10 CPM in a minimum of 15 samples. The filtered miRNA dataset was TMM normalized (see Supplementary Table 1 for normalisation factors), differential expression analysis was performed using edgeR (v3.14.0) and corrected for multiple testing using the Benjamini Hochberg false discovery rate (FDR); FDR adjusted *p*-values < 0.05 were considered statistically significant.

### Validation with RT-qPCR

Identified EV miRNA targets from the small RNA sequencing were validated using RT-qPCR in all 24 patients with COPD and 20 healthy ex-smokers (Figure 1). Detailed methodology can be found in the Supplementary methods (Supplementary material).

### Statistics

Demographics data were analysed by conventional statistical packages (SPSS v27; GraphPad^®^ Prism v9.0). Comparisons between categorical variables were carried out by Chi-squared (if count >5) or Fisher’s exact test (if count ≤5). Shapiro–Wilk test for normality was performed for all continuous variables. Welch two-sample *t*-tests (for normally distributed data) and Mann–Whitney *U* tests (for skewed data) were used to test whether there were significant differences in baseline subject characteristics between COPD subjects and healthy controls. Logistic regression models were used to explore the relationship of co-variables on the proportion of miRNA reads in COPD compared with healthy ex-smokers. Spearman correlation coefficients were generated for non-parametric data. Receiver operative characteristic (ROC) curves were generated using the miRNA normalised expression data in SPSS v27 assuming non-parametric data distribution with 95% confidence intervals. To investigate the predictability of the differentially expressed miRNA to differentiate between health and disease.

## Results

### Subject characteristics

Differential expression analysis was performed on RNA sequencing data from BALF EV miRNA in an initial cohort of 17 patients with COPD and 14 healthy ex-smokers. Confirmation of the RNA sequencing results was performed by RT-qPCR, with an additional 7 COPD patients and 6 healthy ex-smoker controls (who were recruited later to the MICA II study), a total of 44 subjects (subject characteristics summarised in Table 1 and Figure 1). The subjects were matched for age, smoking pack years and body mass index (BMI). As expected, disease defining characteristics such as post-bronchodilator FEV1% predicted, FEV1/FVC ratio, and forced expiratory flow rate (FEF) 25–75% predicted were all significantly reduced in the COPD group. The COPD subjects varied from mild to moderate disease, with a mean FEV1% predicted of 77.5% (SD ± 14.8).

**TABLE 1:** Characteristics of subjects included in the analysis exploring the diagnostic use of bronchoalveolar lavage (BALF) extracellular vesicle (EV) microRNA (miRNA) and associations with inflammatory endotypes, *N* = 44.

Subject/Sample characteristics	COPD (*n* = 24)	Healthy ex-smoker (*n* = 20)	*P*-value
Age, mean ± SD	70.1 ± 6.9	68 ± 7.3	0.34
Male, *n* (%)	20 (83)	11 (55)	0.06
Smoking pack years, mean ± SD	47 ± 29.2	27.8 ± 13	0.06
BMI, mean ± SD	29.6 ± 4	28.4 ± 4	0.3
FEV1 (% predicted), mean ± SD	77.5 ± 14.8	101.8 ± 14.6	< 0.00001
FVC (% predicted), mean ± SD	102.8 ± 16	100.6 ± 16.4	0.65
FEV1/FVC%, mean ± SD	57.7 ± 8.3	78.2 ± 4.2	**<0.00001**
FEF 25–75 (% predicted), mean ± SD	41.2 ± 16.7	106 ± 25.4	**<0.000001**
DLCO (% predicted), mean ± SD	75.1 ± 13.3	88.39 ± 4.4	**0.004**
COPD status, GOLD stage, *n* (%)			0.41
Mild	10 (42)	NA	
Moderate	14 (58)	NA	
**Baseline and historic blood counts**			
Total blood leucocytes (10^9^/L), mean ± SD	7.4 ± 1.4	6.7 ± 1.4	0.09
Absolute neutrophil count (10^9^/L), mean ± SD	4.5 ± 1.2	3.9 ± 1.1	0.12
Absolute eosinophil count (10^9^/L), median (IQR)	0.2 (0.1–0.3)	0.1 (0.1–0.2)	**0.01**
Historic eosinophils (10^9^/L), median (IQR)	0.35 (0.3–0.5)	0.1 (0.1–0.2)	**<0.0001**
**HRCT measurements**			
E/I MLD, mean ± SD	0.85 ± 0.05	0.8 ± 0.05	**0.003**
%LAA_<–950_, mean ± SD	10.9 ± 5.1	6.6 ± 4.5	**0.005**
**BALF differential cell count**			
Neutrophil%, median (IQR)	3.6 (1–9.4)	0.8 (0–1.2)	**0.02**
Macrophage%, median (IQR)	63.7 (35–88.2)	70 (52–80.4)	0.4
Eosinophil%, median (IQR)	1 (0–2.95)	0.4 (0–0.6)	**0.04**
Lymphocyte%, median (IQR)	0 (0–0.55)	0 (0–1.85)	0.08

Fisher’s exact test was performed for gender given small sample size. Chi-squared test used for chronic obstructive pulmonary disease (COPD) status. Shapiro–Wilk test for normality was performed for all continuous variables. Welch two sample *t*-test was performed for normally distributed data: Age, BMI, FEV1, FVC, FEV1/FVC and FEF 25-75, TLCO, RV/TLC SR, total blood leucocytes, absolute neutrophil count, E/I MLD, and %LAA < −950. Mann–Whitney *U* test was performed for skewed data; smoking pack years, eosinophil blood counts and BALF differential cell counts. BMI, body mass index; FEV1, forced expiratory volume in 1 sec, FVC, forced vital capacity; FEF, forced expiratory flow rate; DLCO, diffusion capacity of the lung for carbon monoxide; E/I MLD, ratio of mean lung attenuation on expiratory and inspiratory scans; HRCT, high resolution computer tomography; %LAA < −950, percent of lung voxels on the inspiratory scan with attenuation values below −950 Hounsfield Units; IQR, interquartile range; NA, non-applicable; SD, standard deviation. Bold values represent the statistical significance.

COPD patients had higher levels of historic eosinophil counts than controls (*p* < 0.0001), as defined by highest-ever recorded eosinophil count and higher levels of blood eosinophils at their baseline study enrolment test (*p* = 0.01). When examining the variability of eosinophil counts across the COPD subjects, analysis showed one subject with COPD with higher levels of eosinophils both at baseline (absolute eosinophil count 0.7 10^9^/L, Supplementary Figure 2A) and historically (absolute eosinophil count 1.9 10^9^/L, Supplementary Figure 2B). Interestingly, this was a different COPD subject in each case, demonstrating the variability of eosinophil levels in the blood over time. After excluding these as possible outliers, the significance remained when comparing historic blood eosinophil expression in COPD subjects with health ex-smokers (*p* < 0.001). Furthermore, there were significantly increased proportions of eosinophils in the BALF of COPD subjects compared with the healthy ex-smokers. However, there was no correlation between either baseline eosinophils (*r* = 0.2, *p* = 0.09), nor historic eosinophil counts (*r* = 0.29, *p* = 0.08) and BALF eosinophil expression.

As expected, COPD subjects demonstrated more evidence of small airways disease and emphysema, with a higher ratio of mean lung attenuation on expiratory and inspiratory scans (E/I MLD) (*p* = 0.003), lower diffusion capacity of the lung for carbon monoxide (DLCO) % predicted (*p* = 0.004), and higher percent of lung voxels on the inspiratory scan with attenuation values below −950 Hounsfield Units (%LAA < −950) (*p* = 0.005), compared with healthy ex-smokers.

### Differentially expressed BALF EV miRNA

**FIGURE 2:**
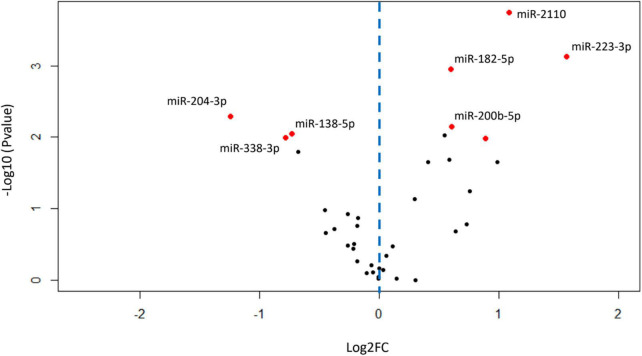
Volcano plot showing relationship between *P*-values and expression data for differentially expressed miRNA validated by RT-qPCR. Red dots show miRNA with *P*-values < 0.05 after false discovery rate (FDR) correction for multiple testing. Blue dotted line represents zero Log2FC, data points to the right are upregulated in chronic obstructive pulmonary disease (COPD), and data points to the left are downregulated in COPD. FC, fold change; miRNA, microRNA; RT-qPCR, real-time quantitative PCR.

Of the 2308 miRNA that were detected in the BALF EVs, 275 miRNAs remained after filtering for low abundance. Of these 275, fifty-four miRNAs were differentially expressed in patients with COPD compared with healthy-ex-smokers in analysis of the RNA sequencing data (Supplementary Figure 3 and Supplementary Table 2) (*n* = 31). Confirmation of these differentially expressed miRNA with RT-qPCR in the larger sample (*n* = 44, see Figure 1) revealed five significantly upregulated miRNA and three significantly downregulated miRNA in patients with COPD compared with healthy controls (Figure 2 and Supplementary Table 3). Of note, miR-625-3p was only detected in the BALF EVs of 18 patients with COPD and 12 healthy ex-smokers.

When comparing types of reads mapped in COPD and healthy ex-smoker samples, there is a higher proportion of miRNA in COPD samples than in healthy ex-smoker samples, both when including unmapped reads (*p* = 0.03) (Supplementary Figure 4A) and without unmapped reads (*p* = 0.02) (Supplementary Figure 4B). Logistic regression was used to look at the effect of co-variables (age, gender, smoking pack year history, and lobe sampled) on the proportion of miRNA reads in COPD compared with healthy ex-smoker samples. The model explained 47–63% (Cox and Snell R2 model–Nagelkerke R2 model) of the variance in COPD and correctly classified 83.9% of cases. Higher miRNA read% was the only variable significantly associated with the presence of COPD (*p* = 0.02) (Supplementary Table 4).

### miRNA expression in COPD phenotypes

Spearman correlation coefficients were generated for the BALF EV miRNA normalised expression data and the clinical phenotypic characteristics of COPD (Table 2). Although significant correlations between clinical variables and EV miRNA expression data were shown when analysing the total cohort (*n* = 44), most of these became non-significant when analysing just the COPD subjects alone (*n* = 24) (Table 2). However, significant correlations were identified for upregulated miR-2110 and miR-200b-5p expression with DLCO% predicted (*r* = −0.43, *p* = 0.04 and *r* = −0.6, *p* = 0.003, respectively), and downregulated miR-338-3p expression with FEF 25–75% (*r* = 0.44, *p* = 0.03), FEV/FVC (*r* = 0.43, *p* = 0.03), and DLCO% predicted (*r* = 0.48, *p* = 0.02) in COPD patients alone (Table 2).

**TABLE 2:** Correlations of lung-derived extracellular vesicle (EV) microRNA (miRNA) expression with chronic obstructive pulmonary disease (COPD) phenotypic disease characteristics.

	FEV1	FVC	FEV1/FVC	FEF 25–75	DLCO	E/L MLD	%LAA_<–950_	Historic Eosinophils (10^9^/L)
**Whole cohort, *N* = 44**								
miR-2110	−0.4**	**−**0.07	−0.46**	−0.47**	−0.37*	0.19	0.26	0.43**
miR-223-3p	–0.26	0.05	−0.42**	−0.44**	−0.38*	0.34*	0.14	0.43**
miR-182-5p	−0.3*	0.05	−0.43**	−0.38*	−0.4*	0.18	0.24	0.32*
miR-625-3p^Γ^	0.005	–0.06	0.007	–0.0005	0.11	–0.05	0.03	0.08
miR-200b-5p	–0.16	0.07	–0.24	–0.23	−0.35*	0.08	0.14	0.28
miR-204-5p	0.35*	–0.13	0.52**	0.47**	0.2	−0.31*	–0.2	−0.33*
miR-138-5p	0.32*	–0.07	0.43**	0.42**	0.28	–0.3	–0.22	−0.35*
miR-338-3p	0.34*	–0.06	0.41**	0.4**	0.4*	–0.26	–0.24	−0.35*
**COPD subjects alone, *N* = 24**								
miR-2110	–0.19	–0.04	–0.12	–0.18	−0.43*	–0.04	0.04	0.01
miR-223-3p	0.02	0.17	–0.07	–0.03	–0.22	–0.03	–0.37	0.02
miR-182-5p	0.04	0.09	–0.07	–0.06	–0.3	–0.25	–0.04	0.03
miR-625-3p^Γ^	0.18	0.01	0.19	0.17	–0.21	–0.07	–0.12	0.1
miR-200b-5p	0.22	0.25	0.07	0.05	−0.6**	–0.05	0.01	–0.04
miR-204-5p	0.28	–0.08	0.36	0.33	0.13	–0.2	–0.05	–0.03
miR-138-5p	0.2	–0.05	0.2	0.26	0.3	–0.2	–0.07	0.008
miR-338-3p	0.29	–0.21	**0.43** *	0.44*	0.48*	–0.25	–0.28	0.004

^Γ^Missing data for 13 COPD subjects, *N* = 11. Spearman’s correlation coefficient.

*p < 0.05, **p < 0.005.

FEV1, FVC, FEF 25–75 and DLCO are all measured as percent predicted. Historic eosinophil refers to highest ever recorded eosinophil count. FEV1, forced expiratory volume in 1 sec, FVC, forced vital capacity; FEF, forced expiratory flow rate; DLCO, diffusion capacity of the lung for carbon monoxide; E/I MLD, ratio of mean lung attenuation on expiratory and inspiratory scans; %LAA < −950, percent of lung voxels on the inspiratory scan with attenuation values below −950 Hounsfield Units. Bold values represent the statistical significance.

### Predictive capacity of EV miRNA

Given the lung-derived EV miRNA were associated with many of the clinical phenotypic characteristics across the cohort, the predictive ability of the upregulated miRNA to differentiate between health and COPD was assessed. Only upregulated miRNA were chosen, as if future work developed EV miRNA as a biomarker of early disease, one would look for presence of a marker in disease rather than absence.

**FIGURE 3:**
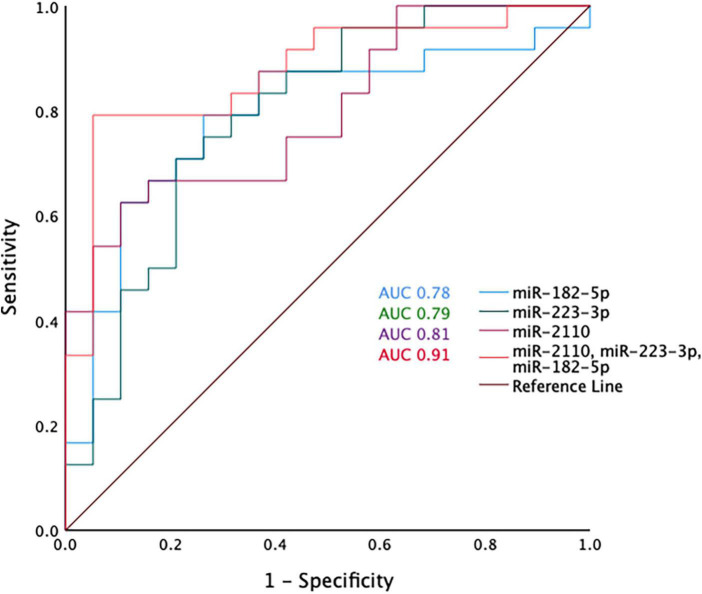
Receiver operator curve analysis for miR-2110, miR-223-3p, and miR-182-5p and the combination of miR-2110, miR-223-3p, and miR-182-5p.

Receiver operative characteristic (ROC) curves were generated using the miRNA normalised expression data and showed that individually miR-2110, miR-223-3p and miR-182-5p have moderate predictive ability to differentiate between COPD and healthy ex-smokers, with an area under the curve (AUC) > 0.7 (Figure 3 and Supplementary Table 5). Although miR-625-3p performed nearly as well, this was excluded from further analysis as it was only found expressed in a sub-cohort of subjects (*N* = 33, *n* = 18 COPD) (Supplementary Table 5). The combination of miR-2110, miR-223-3p and miR-182-5p improved the predictive ability to discriminate between COPD and healthy ex-smokers, with an AUC 0.91 (Figure 3).

### miRNA in COPD inflammatory endotypes

There were significantly increased proportions of neutrophils and eosinophils in the BALF of COPD subjects compared with the healthy ex-smokers (Table 1). Macrophages were the predominant cell type in the airways (median proportion 68%), however, there was no difference in macrophage proportions between COPD subjects and healthy ex-smokers (Table 1, *p* = 0.4).

The relationship between BAL EV miRNA expression and levels of inflammatory cells was assessed in the COPD subjects alone (*N* = 24), (Supplementary Table 6). There were significant positive correlations between levels of neutrophils and two of the upregulated EV miRNA in COPD (miR-2110 and miR-182-5p). Whereas miR-223-3p significantly correlated with levels of eosinophils (*r* = 0.47, *p* = 0.03). Conversely, in the downregulated EV miRNA, miR-204-5p showed significant negative correlations with both neutrophils and eosinophil expression, whereas miR-338-3p only significantly correlated with eosinophils (*r* = −0.42, *p* = 0.03).

The significant correlation with miR-223-3p, miR-204-5p and miR-338-3p with BALF eosinophil levels prompted further analysis with blood eosinophil levels given the clinical utility of historic blood eosinophil count in defining eosinophilic disease in COPD. However, there was no correlation between BALF eosinophil levels and historic blood eosinophil count (*r* = 0.1, *p* = 0.65). Furthermore, there was no association between historic eosinophil count and miR-223-3p (*r* = 0.02, *p* = 0.9), miR-204-5p (*r* = −0.03, *p* = 0.9) and miR-338-3p (*r* = 0.004, *p* = 0.9) expression levels in the lung-derived EVs.

**FIGURE 4:**
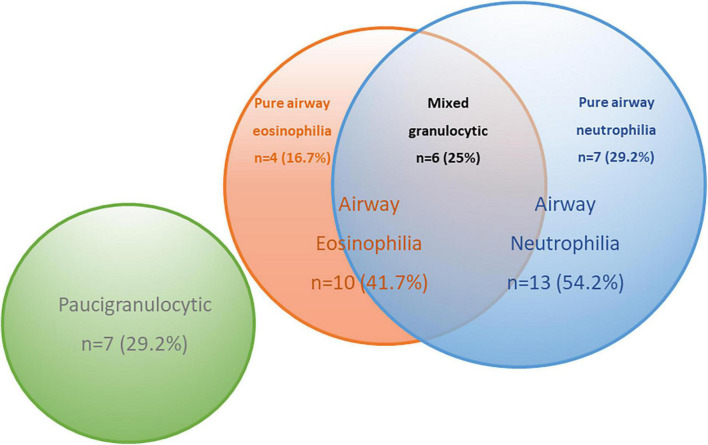
Venn diagram to describe the inflammatory endotypes in the chronic obstructive pulmonary disease (COPD) subjects based on pre-defined cut-offs.

The significant correlations between levels of BALF inflammatory cells and specific EV miRNA in COPD subjects raised the possibility of EV miRNA ability to predict specific inflammatory endotypes in COPD. COPD subjects were split into inflammatory endotypes based on American Thoracic Society defined cut-offs (25) (Supplementary Table 7). The 24 COPD subjects were classified as eosinophilic (>1% eosinophils, *n* = 10), neutrophilic (>3% neutrophils, *n* = 13), mixed granulocytic (>1% eosinophils and >3% neutrophils, *n* = 6), or paucigranulocytic (≤1% eosinophils and ≤3% neutrophils, *n* = 7) (Figure 4).

A series of ROC analyses were performed to determine the predictive ability of miRNA to determine inflammatory endotypes. Firstly, the eosinophilic subjects with COPD (*n* = 10) were compared against the non-eosinophilic COPD subjects (*N* = 14; pure airway neutrophilia, *n* = 7 and paucigranulocytic, *n* = 7). MiR-223-3p and miR-338-3p showed good predictive ability to distinguish between eosinophilic and non-eosinophilic disease (AUC > 0.7, *p* < 0.05), however, combining these measures, the AUC improved to 0.83 (*p* = 0.007) (Table 3).

**TABLE 3:** Receiver operative characteristic (ROC) analyses to determine predictive ability of miRNA to differentiate between eosinophilic and non-eosinophilic subtypes in chronic obstructive pulmonary disease (COPD).

miRNA	AUC (95% CI)	Standard Error^a^	*P*-value
**Airway eosinophilia (*n* = 10) versus pure airway neutrophilia and paucigranulocytic (*n* = 14)**			
miR-2110	0.51 (0.2–0.8)	0.14	0.9
**miR-223-3p**	**0.78** (0.6–1)	0.14	**0.04**
miR-182-5p	0.57 (0.3–0.8)	0.14	0.6
miR-625-3p^Γ^	0.30 (0.04–0.6)	0.14	0.14
miR-200b-5p	0.51 (0.2–0.8)	0.14	0.9
miR-204-5p	0.74 (0.5–0.9)	0.05	0.05
miR-138-3p	0.60 (0.4–0.8)	0.41	0.4
**miR-338-3p**	**0.74** (0.5–0.9)	0.05	**0.046**
**miR-223-3p, miR-338-3p**	**0.83** (0.7–0.9)	0.12	**0.007**
**Pure airway eosinophilia (*n* = 4) versus pure airway neutrophilia and paucigranulocytic (*n* = 14)**			
miR-2110	0.75 (0.3–1)	0.21	0.25
**miR-223-3p**	**0.94** (0.8–1)	0.09	**0.04**
miR-182-5p	0.81 (0.5–1)	0.18	0.15
miR-625-3p^Γ^	0.75 (0.4–1)	0.19	0.25
miR-200b-5p	0.88 (0.6–1)	0.14	0.08
miR-204-5p	0.86 (0.6 -1)	0.13	0.06
miR-138-3p	0.68 (0.3–1)	0.18	0.35
**miR-338-3p**	**0.85** (0.6–1)	0.08	**0.03**
**miR-223-3p, miR-338-3p**	**0.81** (0.6-1)	0.09	**0.04**

^a^Standard error under the non-parametric assumption.

^Γ^Data missing for 13 subjects.

Bold values represent the statistical significance.

Given the combination of miR-223-3p and miR-338-3p showed good predictive ability in distinguishing eosinophilia when including subjects with a mixed granulocytic picture; further analysis was performed to see whether these miRNAs were even more specific at distinguishing eosinophilic disease when considering just pure eosinophilic disease (*n* = 4). MiR-223-3p showed excellent predictive ability of differentiating pure airway eosinophilia from paucigranulocytic and pure airway neutrophilic disease with an AUC 0.94 (*p* = 0.04). MiR-338-3p was also significant in distinguishing pure airway eosinophilia with an AUC 0.85 (*p* = 0.03). The combination of the two miRNAs did not improve the specificity with an AUC 0.81 (Table 3).

Finally, the neutrophilic subjects with COPD (*n* = 7) were compared against the non-neutrophilic COPD subjects (*N* = 11; pure airway eosinophilia, *n* = 4 and paucigranulocytic, *n* = 7). However, none of the miRNA showed a significant predictive ability for distinguishing between neutrophilic and non-neutrophilic disease (Supplementary Table 8).

## Discussion

We present the first analysis of differentially expressed EV miRNA in BALF of patients with mild-moderate COPD compared to healthy ex-smokers. Results from the ROC curve analysis demonstrated that the combination of miR-2110, miR-223-3p and miR-182-5p had excellent predictive ability (AUC 0.91) in discriminating between COPD and healthy ex-smokers. Importantly this was shown in a relatively mild COPD cohort. Currently the diagnosis of COPD depends on the use of spirometry to define airflow obstruction, however, using this measure alone may fail to detect early-stage disease (e.g., GOLD stage 0 disease). Consequently, having a more sophisticated biomarker of disease that can detect pre-clinical disease could have significant implications for risk-factor modification, treatment initiation and long-term prognosis.

MicroRNA are posed as ideal biomarker candidates as they are easily measurable in liquid biopsies (e.g., blood, urine, sputum, and BALF) and have demonstrated high sensitivity for differentiating stages of disease and even treatment responsiveness (26). Urinary exosomal miRNA have been shown to detect early renal fibrosis in lupus nephritis (27) and a nine-miRNA multimarker panel for breast carcinoma has been shown to significantly improve reliability of breast cancer diagnosis (28). Furthermore, the technologies for detection of these small non-coding RNAs are advancing at speed with the development of newer assays requiring less time and lower costs in comparison to producing new antibodies for protein biomarkers.

Importantly, our results show a higher proportion of miRNA in COPD BALF EVs than healthy ex-smokers. To our knowledge, only one other study has previously shown altered proportions of miRNAs in EVs in disease. Francisco-Garcia et al. showed deficient loading of miRNAs in BALF EVs of severe asthmatics compared with healthy controls. In addition, pathway analysis suggested that these significantly downregulated miRNAs in severe asthmatics converge on pathways known to be important in asthma pathogenesis (29). Cells can selectively sort miRNA into EVs for secretion to nearby or distant targets. Broadly these mechanisms include RNA-binding proteins such as hnRNPA2B1, membranous proteins involved in EV biogenesis such as nSMase2, and specific miRNA-binding motifs capable of exerting selectivity over the miRNAs shuttled into EVs (30). Current EV miRNA literature focuses on the dysregulated EV-miRNA content; however, little is known about the role of disease pathogenesis in regulating the EV miRNA selective sorting process. Therefore, understanding the sequences and/or proteins responsible for selective sorting of miRNA in COPD lung-derived EVs may reveal novel mechanisms in the disease pathogenesis, and provide targets for manipulating EV content that could have beneficial disease modifying effects.

Bronchoscopy is an invasive procedure with limits on sample availability and in particular reference to biomarker discovery, we recognise that sampling of more accessible biofluids such as blood, sputum or exhaled breath will be key to determining the utility of EV miRNA as biomarkers in future. Endothelial microparticles have been analysed in both blood (21) and sputum (31) as possible biomarkers of stable COPD, and Tan et al. (32) showed that levels of exosomal EVs were higher in patients with an acute exacerbation of COPD than stable disease. However, further work is required to explore the EV miRNA signature in COPD, with a focus on cell/tissue specific surface marker identification to increase its utility and specificity especially in compartments (e.g., blood) that may reflect other co-existing multimorbid conditions.

Correlative analysis showed there were significant positive correlations between neutrophil expression and miR-2110 and miR-182-5p; and eosinophil expression and miR-223-3p and miR-338-3p. Whereas miR-204-5p showed significant negative correlations with both neutrophil and eosinophil expression. These associations raise questions about the origin of these lung-derived EVs and their possible target cells. For example, a positive correlation may suggest that a specific cell type (e.g., neutrophil) may be the dominant source of a particular EV miRNA (e.g., miR-182-5p) or recruited as a result of high expression.

Exploring the mechanisms of miRNA regulation of neutrophil function in COPD may provide key insights into neutrophil dysfunction and identify alternative targets for treatment. MicroRNA-182-5p is already known to regulate neutrophils, with Li et al. (33) showing that miR-182-5p enhances neutrophil migration into the vascular endothelium. In addition, miR-182 has been shown to regulate granulopoiesis via inhibition of C/EBPα (a master regulator of granulopoiesis) suggesting a role in neutrophil generation (34). To date, this present study is the first to link miR-2110 to neutrophil accumulation in the airways, where previous work has focused solely on its role in tumorigenesis (35).

A previous study has shown that miR-204-5p inhibits inflammation and chemokine generation in renal tubular epithelial cells by modulating IL-6 expression (36), where IL-6 is an important regulator of neutrophil recruitment in response to lung inflammation (37). Thus, downregulation of lung-derived EV miR-204-5p in the COPD lung could lead to increased neutrophils via an IL-6-dependant pathway. It is tempting to think that novel therapeutics targeting this pathway could reduce excessive airway neutrophilia, as well as prevent airway inflammation and tissue destruction.

In this study, eosinophil expression was shown to significantly correlate with miR-204-5p, miR-223-3p and miR-338-3p expression. Furthermore, miR-223-3p and miR-338-5p showed good predictive ability at identifying airway eosinophilia (>1% eosinophils). Upregulation of non-EV miR-223-3p has been reported in COPD, both in lung tissue compared with smokers (38) and BALF cell pellets (39). Moreover, miR-223-3p levels in bronchial biopsies was previously shown to correlate with eosinophils in asthmatics (40), and are significantly increased in patients with allergic rhinitis (41), where miR-223 is shown to enhance eosinophilic infiltration (42). Together these findings suggest that both these miR-223-3p and miR-338-5p may play a role in defining eosinophilic airways disease in COPD, however, the underlying mechanisms are yet to be clearly defined. Previous work by Asensio et al. found that miR-619-5p and miR-4486 were differentially expressed in the serum of COPD patients with eosinophilia (43). However, circulating miRNA may have a different cellular origin and function to miRNA within lung EVs, and although EVs are an obvious vehicle for miRNA transfer from the lungs into the peripheral circulation, future work identifying lung specific EVs in blood is an opportunity to explore their utility as a biomarker of disease and relationship with eosinophilia.

There has been considerable interest in the role of blood eosinophil counts in predicting treatment responsiveness to corticosteroids in COPD patients, based on the premise that they reflect and correlate with tissue eosinophilic inflammation (7, 44). However, in this study, blood eosinophilia did not correlate with BAL eosinophilia, and further analysis of historic blood eosinophil expression in COPD patients showed no relationship with lung EV miR-223-3p, miR-204-p and miR-338-3p expression. This is in keeping with more recent work which suggests that blood eosinophils do not correlate with lung tissue eosinophilia (45). Defining eosinophilic inflammation in COPD is challenging, with numbers of eosinophils differing during stable disease, exacerbations and following treatment (46), with blood eosinophil counts known to fluctuate in individuals during a 24-h period (47). Furthermore, although eosinophilic promoters, such as IL-5, are increased in patients with eosinophilic COPD (48), targeted eosinophilic treatments (such as anti-IL-5 therapies) have had limited success thus far (11). Therefore, exploring novel mechanisms for airway eosinophilia in COPD, possibly through an EV miRNA mechanism, could provide new therapeutic targets.

We recognise the main limitation of this study is the small sample size and associated limited statistical power particularly when analysing the inflammatory subgroups. However, the sample size is comparable to other studies using human BALF samples to explore EV miRNA differences (49, 50) and any differences in EV miRNA expression were corrected for multiple testing. We acknowledge the limitations of performing this analysis in the same cohort as the discovery samples and therefore the high AUC reported may be a product of over-fitting. In addition, we acknowledge the cross-sectional sampling approach does not allow us to assess whether these differences in EV miRNA expression would be stable over time. As mentioned previously, previous work has shown plasma EV levels may vary in disease state (32), however, to our knowledge nothing is known of the stability of EV miRNA expression over time. Despite the small sample groups, the extensive characterisation of the subjects allowed exploration into the association of the differentially expressed lung EV miRNA with different subgroups of disease. These findings are promising for discovery of new inflammatory endotypes in COPD and possible identification of new targets for precision-based medicine.

As previously discussed, the COPD patients included in this study had relatively mild disease. This contrasts with other EV miRNA studies in COPD (20, 21, 31), which included a broader range and severity of COPD patients (mean FEV1 63.4%, SD ± 29.54) and current smokers. Although their findings may be applicable to a wider COPD cohort, they are less translatable mechanistically given their broader range of included subject phenotypes and the inclusion of current smokers, which may attribute effects to active smoking rather than disease alone.

To our knowledge, this is the first study to identify differentially expressed miRNA in BALF in patients with COPD. These findings suggest specific lung-derived EV miRNA are a strong predictor of disease presence in COPD, even in mild disease. Further work should be directed into whether these findings can be translated into other accessible biofluids to increase their utility as a diagnostic biomarker. We further demonstrate that specific lung EV miRNA correlate with neutrophilic and eosinophilic COPD, highlighting the potential utility of this approach in defining inflammatory endotypes, which could be important in future treatment stratification.

## Data availability statement

The data presented in this study are deposited in the GEO repository, accession number: GSE218571.

## Ethics statement

The studies involving human participants were reviewed and approved by National Research Ethics Service South Central Ethical Standards–Hampshire A and Oxford C Committees (LREC no: 15/SC/0528). The patients/participants provided their written informed consent to participate in this study.

## Author contributions

HB and TW conceptualized the project. HB, AW, and CS contributed to the methodology. HB undertook the formal analysis, performed the investigation, curated the data and wrote the original draft, and had full access to the data in the study and takes responsibility for the integrity of the data. HB, AW, DC, AF, NW, AH, KO, CS, and KS administered the project. CS, KS, and TW supervised the project. All authors contributed to the writing, reviewing and editing, and approved the final manuscript.
