# Supplementary material for: Pulmonary EV miRNA profiles identify disease and distinct inflammatory endotypes in COPD
Source: Front Med (Lausanne). 2022 Dec 15;9:1039702. doi: 10.3389/fmed.2022.1039702 (PMC9797812; doi:10.3389/fmed.2022.1039702)
Supplement: Supplementary file 1 [file Data_Sheet_1.PDF]

Supplementary Material

1 Appendix 1:

MICAII study group

Bastian Angerman 1

Stephanie Ashenden 2

Sarah Bawden 3

Graham Belfield 2

Maria G. Belvisi 1,4

Aurelie Bornot 2

Jerome Bouquet 5

Hannah Burke 3, 6

Carolina Caceres 5

Raghothama Chaerkady 7

Doriana Cellura 3, 6

Chia-Chien Chiang 8

Kerry Day 3, 6

Antonio DiGiandomenico 5

Hanna Duàn 1

Ulrika Edvardsson 9

Damla Etal 2

Anna Freeman 3, 6

Matthew S. Glover 7

Vancheswaran Gopalakrishnan 5

Stephen Harden 10

Sonja Hess 7

Alex Hicks 3, 6

Ventzislava A. Hristova 7

Michael Hühn 1

Fredrik Karlsson 2

Shameer Khader 8

Glenda Lassi 1

Alex Mackay 1,4

Christopher McCrae 1

Christopher Morehouse 5

Daniel Muthas 1

Karl Nordström 2

Steven Novick 2

Esther Nyimbili 3

Kristoffer Ostridge 1, 6

Lisa Öberg 1

Adam Platt 14

Laura Presland 3

Xiaotao Qu 8

Nicola Rayner 3

Pedro Rodrigues 3

Bret Sellman 5

Gary Sims 1

Cosma Mirella Spalluto 6

Andria Staniford 3

Karl J. Staples 3, 6

Bruce Thompson 12

Outi Vaarala13

Junmin Wang 7

Paul Warrener 5

Alastair Watson 6

Nicholas P. Williams 3, 6

Tom M. A Wilkinson 3, 6

Wen Yu 8

Bairu Zhang 2

Tianhui Zhang 2

Natalie van Zuydam 2

1 Research and Early Development, Respiratory & Immunology, BioPharmaceuticals R&D, AstraZeneca, Gothenburg, Sweden

2 Translational Genomics, Discovery Biology, Discovery Sciences, BioPharmaceuticals R&D, AstraZeneca, Gothenburg, Sweden

3 NIHR Southampton Biomedical Research Centre, Southampton, UK

4 National Heart & Lung Institute, Imperial College London, London, UK

5 Microbial Sciences, BioPharmaceuticals R&D, AstraZeneca, Gothenburg, Sweden

6 Faculty of Medicine, University of Southampton, Southampton, UK

7 Dynamic Omics, Centre for Genomics Research, Discovery Sciences, BioPharmaceuticals R&D, AstraZeneca, Gaithersburg, USA

8 Data Science and Artificial Intelligence, BioPharmaceuticals R&D, AstraZeneca, Gothenburg, Sweden

9 Business Development and Licensing, BioPharmaceuticals R&D, AstraZeneca, Gothenburg, Sweden

10 University Hospital Southampton NHS Foundation Trust, Southampton, UK

11 Research and Early Development, Respiratory & Immunology, BioPharmaceuticals R&D, AstraZeneca, Gaithersburg, USA

12 Swinburne University of Technology Melbourne, Australia

13 Faculty of Medicine, University of Helsinki, Helsinki, Finland

14 Research and Early Development, Respiratory & Immunology, BioPharmaceuticals R&D, AstraZeneca, Gaithersburg, USA

2 Supplementary Methods

2.1 Bronchoalveolar lavage fluid analysis

BAL fluid was poured through 100 µm cell strainer to remove mucus and cells were removed by centrifugation at 400 g, 4oC for 10 min. The cell-free supernatant was stored at -80ºC prior to EV isolation, while the resulting cell pellet was resuspended in 10 mL hypotonic lysis buffer for 2 min to remove any red blood cell contamination. Ten millilitres of hypertonic recovery buffer was then added with 1X PBS to make-up the volume to 40 mL. The sample was centrifuged at 400 g for 10 min. The pellet was then resuspended in 1 mL 1X PBS and cell counts were performed using the Trypan blue exclusion method. The cell solution was subsequently adjusted to 0.5 x 106 cells/mL, and 75 µL of this solution was loaded onto cytospin funnels. Cells were centrifuged at 350 g for 6 min and collected on Poly-L-Lysine slides.

Cell slides were left to air-dry overnight and stained the next day using Rapid Romanowsky A-B-C kit (TCS Biosciences, Buckingham, UK). A differential cell count was performed by counting 500 cells using light microscopy.at X40 magnification. Numbers and relative percentages of eosinophils, neutrophils, macrophages, lymphocytes, bronchial epithelial cells and squamous cells were calculated.

2.2 Extracellular vesicle isolation and characterisation

Fifteen millilitres of BALF were centrifuged at 400g for 10 min to remove cell debris, filtered through a 0.22 µm PVDF, 33 mm gamma sterilised filter (Merck Millipore®, Watford, UK) to remove larger particles (e.g. apoptotic bodies), and then concentrated using a Amicon® Ultra-15 (10,000 MWCO) spin filter according to manufacturer's instructions. The resultant ~2mL EV containing sample passed through a size exclusion chromatography platform (PURE-EV™, HansBioMed®, Tallinn, Estonia) under gravity, with the addition of 10mL of 1X Phosphate-Buffered Saline (PBS). Twenty-four 500 µL fractions were collected and fractions 6-11 were collated containing the purified EVs.

The purified EV population were characterised using a CD9 double sandwich enzyme-linked immunosorbent assay (CD9 ELISA, ExoTest™, HansaBioMed®) using manufacturer's instructions. Transmission electron microscopy with negative staining was used to visualise the characteristic EV cup-shaped morphology and size. SDS PAGE and Western blotting were used to determine the presence of CD63 and absence of the endoplasmic reticulum marker calnexin, from the isolated BALF EVs compared with the BALF cellular pellet. Briefly EVs were lysed and denatured in NuPAGE™ LDS Sample buffer 4X with 12% B-Mercaptoethanol (ThermoFisher Scientific®) at 70°C for 10 min and resolved in NuPAGE™ 4-12% Bis-Tris Protein gels (ThermoFisher Scientific®). Expression of CD63 (anti-rabbit polyclonal antibody, Atlas Antibodies, Bromma, Sweden) and calnexin (anti-rabbit monocolonal antibody, Cell Signaling Technology, Danvers, US) were detected by specific antibodies.

2.3 EV RNA isolation, library preparation and smallRNA sequencing of BALF EVs performed by QIAGEN® Genomic Services

Prior to RNA isolation, BALF EVs (suspended in 200 µL of 1X PBS) were lysed using Buffer RPL at room temperature for 3 min. To assess the quality of RNA isolation across samples, Qiaseq miRNA Library Quality control (QC) Spike-Ins solution (containing 52 Qiaseq miRNA library QC spike ins) was added to each of the lysed EV samples. RNA from EVs was then isolated by Qiagen®

using the miRNeasy® Serum/Plasma Advanced kit (Qiagen®) following the manufacturer's instructions.

Reverse transcription was performed in 10 µL reactions using the miRCURY LNA RT kit (Qiagen®), with an artificial RNA spike-in (UniSp6) to assess the quality of the reverse transcriptase reaction.

Following cDNA synthesis, quantitative PCR was performed by Qiagen in a LightCycler® 480 Real-Time PCR System (Roche®, Welwyn Garden City, UK) in 384 well plates. Primers used were: miR-23a, miR-30c, miR-103, miR-142-3p and miR-451, as well as the primers for the 52 RNA spike-ins (listed in Table 2.3) and the primer for the artificial RNA spike-in (UniSp6). Negative controls excluding the template from the reverse transcription reaction was performed and profiled like the samples. Amplification curves were analysed using the Roche® LC software, both for determination of Cq (by the 2nd derivative method) and for melting curve analysis.

Library preparation was performed using Qiaseq™ miRNA Library Kit (Qiagen®) according to the manufacturer's instructions. Due to the low input of RNA (1ng), the Qiaseq miRNA NGS 3' Adapter was diluted 1:10 with nuclease-free water. The quantity and quality of miRNA libraries were determined using a high sensitivity DNA chip on an Agilent® Bioanalyser 2100. The concentration of the library was quantified on a Qubit™ Fluorimeter (ThermoFisher Scientific). The individual libraries were diluted with nuclease-free water to 4 nM. The libraries were combined in equimolar amounts ready for sequencing. A final concentration of 1.8 pM of the denatured, diluted library solution was sequenced on the NextSeq500 instrument (Illumina®, Chesterford, UK). Average reads per sample were 2.8 million.

2.4 Trimming of adaptors and UMI correction performed by Qiagen® Genomic Services

Sequencing data was demultiplexed, and BCL files converted to standard FASTQ file format for downstream analysis using bcl2fastq conversion software v2.20 (Illumina®). Next, cutadapt (v1.11) is used to remove low quality bases and identify the adapter and UMIs applied during library. The output from cutadapt is used to remove adapter sequences and to collapse reads by unique molecular index (UMI) with in-house script. Each raw read is expected to contain (starting from the 5' end): an insert sequence, the adapter sequence, 12nt-long UMI sequence, and other ligated sequence. Only reads that contain adapters, have insert sequences ≥16 nucleotides and a UMI length ≥10 nucleotides are kept. Insert sequences from reads which do not contain full length UMI sequence are output as "partial-UMI reads". Full IMU length reads with identical insert and UMI sequences are merged and then combined with partial-UMI reads as output of UMI correction (summarized in flowchart below).

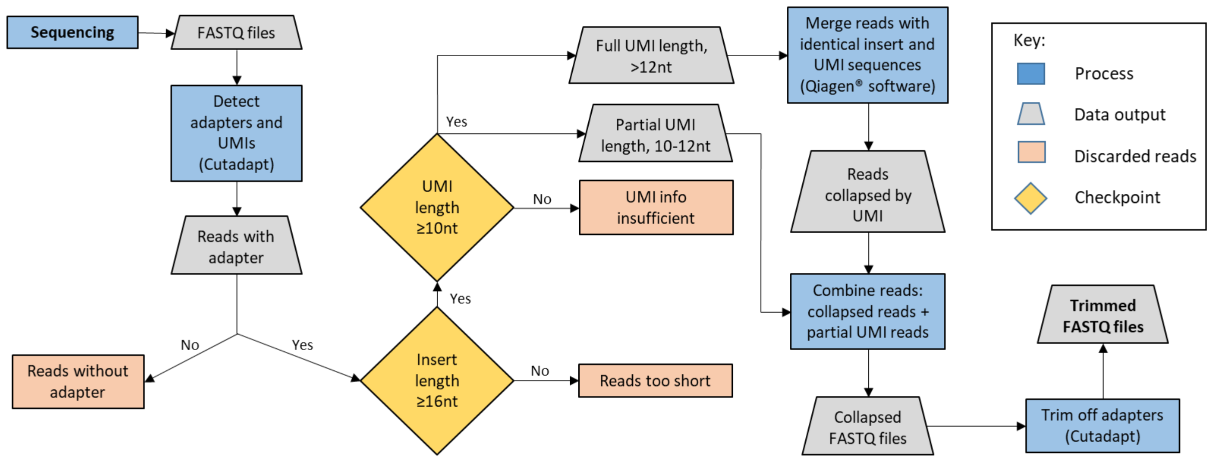

2.5 miRNA sequencing quality control, mapping and alignment

Trimmed FASTQ files were analysed for read quality using FastQC tool. Bowtie2 (v 2.2.2) tool was used to align sequencing reads to the reference genome (GRCh37/hg19), and miRNA to the miRNA database, miRBase (version mirbase_20).

2.6 miRNA differential expression analysis

Differential gene expression were assessed with edgeR (v3.14.0) in R (v 3.8.2). Firstly, lowly expressed miRNA (CPM <10 in 15 samples) were filtered out of the dataset. Reads underwent normalisation using the calcNormFactors function in edgeR which normalises for RNA composition by finding a set of scaling/normalisation factors for the library sizes that minimise the log-fold changes between the samples for most miRNA. The default method for computing these scale factors uses a trimmed mean of M-values (TMM) between each pair of samples. The normalisation factors of all the libraries multiply to unity. The recalculated library sizes and normalisation factors are listed in a Table S1. The TMM-normalised dataset was then used for the differential expression analysis between patients with COPD and healthy ex-smokers.

The following code performs the TMM normalisation and generates a list of normalisation factors for each sample, where "keep" is the log-transformed CPM dataset with the lowly expressed miRNA removed.

```
> TMM_normalised <- calcNormFactors(keep)

> TMM_normalised$samples
```

Negative binomial distribution methods were used to model the TMM normalized dataset. The following code was used to calculate dispersion estimates where "TMM_normalised" is the TMM normalised miRNA dataset and "design" is a model matrix based on the experimental design of the study (i.e. samples either assigned to COPD or Healthy).

```
> Condition <- factor(group[ ,"Disease"], levels=c("Healthy", "COPD")

> design <- model.matrix(~Condition)

> y <- estimateDisp(TMM_normalised,design)
```

```
## The square root of the common dispersion gives the BCV

> sqrt(y$common.dispersion)
```

The common dispersion estimate was calculated as 0.398 (~0.4 is usual for biological studies). Trended dispersion estimates and miRNA specific estimates (referred to as "Tagwise" in edgeR) were used in testing for differential expression. Once negative binomial models were fitted and dispersion estimates obtained, edgeR determined differential miRNA expression using the exact test. The following code was used to determine differential miRNA expression, where "y" is the dispersion estimates calculated above:

```
>  et <- exactTest(y)

## To give the top 10 differentially expressed miRNA

> topTags(et)

## To list the miRNA differentially expressed at a false discovery rate (FDR) of 5%

> results_edgeR <- topTags (et, n= nrow(data_clean), sort.by = "none")

> sum(results_edgeR$table$FDR<0.05)

## To visualise the data on an MA plot, showing the log2 fold change on y axis versus average log 2 CPM on x axis for differentially expressed miRNA, with miRNA with an FDR<0.05 in red.

> plotSmear (et, de.tags  = rownames (results_edgeR) [results_edgeR$table $FDR<0.05], pch=16, cex=1)

## Additional information can be added to the MA plot e.g. blue line representing two-fold change in expression

> abline(h=c(-1,1), col = "blue")
```

2.7 RT-qPCR confirmation of RNA sequencing results

To confirm the findings of the BALF EV RNA sequencing results, RT-qPCR was performed on 44 BALF EV samples for a total of 46 assays, including 6 stably expressed miRNA for normalization and 5 RNA spike-ins to assess RNA isolation efficiency and the quality of the reverse transcription reaction.

NormFinder software in Microsoft® Office Excel was used to identify miRNA that were most stably expressed across all samples from the miRNA sequencing results (ref). These miRNA were then used as normalisers for the qPCR validation study. Although all of these "normaliser" miRNA were measured by RT-qPCR, only "normaliser" miRNA detected in all samples were used for normalisation of Cq data.

Total RNA was isolated from BALF EVs as stated previously. Reverse transcription was performed in 10µL reactions using the miRCURY LNA RT kit (Qiagen®). Following cDNA synthesis, qPCR was performed in a LightCycler® 480 Real-Time PCR System (Roche®) in 384 well plates. Amplification curves were analysed using the Roche LC software, for both determination of Cq (by the 2nd derivative method) and for melting curve analysis. The mean Cq for all the universally

expressed “normaliser” miRNA was calculated to give a Geomean Cq. Then, the following formula was used to calculate the normalized Cq values:

Normalized Cq of miRNA of interest (ΔCq) = Geomean Cq – miRNA of interest Cq

A higher value thus indicates that the miRNA is more abundant in the particular sample. Values were then presented as 2ΔCq to represent fold change.

3 Supplementary Tables

Table S1. List of library sizes and normalization factors generated by TMM normalization

Sample ID	Sample cohort	Adjusted library size	Normalisation factor
MICA_II_077_RML	Healthy	193077	0.996
MICA_II_078_RML	COPD	1092789	1.000
MICA_II_061_RLL	Healthy	175842	1.046
MICA_II_065_LLL	Healthy	294122	0.977
MICA_II_109_RML	Healthy	601252	0.985
MICA_II_141_RML	Healthy	659567	0.985
MICA_II_128_RLL	Healthy	214688	0.970
MICA_II_130_LLL	Healthy	496871	1.004
MICA_II_126_RML	COPD	1248508	1.106
MICA_II_095_LLL	Healthy	768741	0.922
MICA_II_085_RUL	COPD	371065	0.919
MICA_II_094_LLL	COPD	364761	0.901
MICA_II_132_RML	COPD	1805946	1.007
MICA_II_081_RML	COPD	801747	1.128
MICA_II_105_RML	COPD	844511	0.969
MICA_II_046_RUL	Healthy	343816	0.899
MICA_II_104_RML	COPD	1152597	0.990
MICA_II_064_RML	COPD	1089079	0.974
MICA_II_056_RLL	Healthy	313940	1.034
MICA_II_062_RML	Healthy	344192	1.067
MICA_II_076_LUL	Healthy	333900	1.008
MICA_II_082_LLL	COPD	732867	1.007
MICA_II_131_RML	Healthy	127769	1.017
MICA_II_079_RML	COPD	810943	1.072
MICA_II_097_RUL	COPD	1077755	0.974
MICA_II_134_LLL	Healthy	699110	0.961
MICA_II_069_RML	COPD	1076087	1.161
MICA_II_093_RML	COPD	373396	0.996
MICA_II_098_RML	COPD	678973	1.046
MICA_II_133_RML	COPD	882149	0.935
MICA_II_147_LLL	COPD	451583	0.998

Table S2. Differentially expressed EV miRNA identified by RNA sequencing

Assay	Log2FC	logCPM	P Value	FDR
**Upregulated in COPD based on sequencing results**				
hsa.miR.223.3p	1.73	10.87	<0.0001	0.006
hsa.miR.20b.5p	1.03	4.79	0.010	0.048
hsa.miR.629.5p	0.92	5.74	0.0004	0.010
hsa.miR.31.5p	0.88	9.64	0.002	0.019
hsa.miR.625.5p	0.87	4.54	0.0018	0.019
hsa.miR.574.5p	0.83	6.59	0.006	0.038
hsa.miR.2110	0.83	8.15	0.0001	0.006
hsa.miR.185.5p	0.77	8.04	0.0005	0.011
hsa.miR.769.3p	0.77	4.54	0.004	0.030
hsa.miR.146a.5p	0.73	12.40	0.005	0.034
hsa.miR.191.5p	0.68	13.49	0.0002	0.006
hsa.miR.25.5p	0.66	4.76	0.003	0.022
hsa.miR.345.5p	0.64	7.36	0.002	0.019
hsa.miR.589.5p	0.62	4.48	0.004	0.031
hsa.miR.200b.5p	0.56	8.87	0.0001	0.006
hsa.miR.625.3p	0.54	7.27	0.003	0.026
hsa.miR.182.5p	0.53	11.04	<0.0001	0.006
hsa.miR.941	0.47	6.71	0.002	0.019
hsa.miR.7.5p	0.46	7.15	0.007	0.042
hsa.miR.375	0.45	13.35	0.002	0.019
hsa.miR.183.5p	0.43	9.90	<0.0001	0.006
hsa.miR.320a	0.42	10.97	0.002	0.019
hsa.miR.324.5p	0.42	6.36	0.0018	0.019
hsa.miR.151a.5p	0.30	7.24	0.007	0.042
hsa.miR.151a.3p	0.25	11.26	0.005	0.036
**Downregulated in COPD based on sequencing results**				
hsa.miR.204.5p	-1.36	6.97	0.002	0.019
hsa.miR.338.3p	-1.18	10.65	0.0002	0.007
hsa.miR.138.5p	-1.02	6.45	0.0002	0.007
hsa.miR.138.1.3p	-0.98	4.53	0.008	0.046
hsa.miR.30d.3p	-0.96	6.85	0.0008	0.013
hsa.miR.934	-0.88	5.03	0.0006	0.011
hsa.miR.30b.5p	-0.82	11.31	0.0008	0.013

hsa.miR.181c.5p	-0.81	4.67	0.0020	0.019
hsa.miR.17.3p	-0.77	4.23	0.010	0.048
hsa.miR.1277.5p	-0.77	5.23	0.009	0.047
hsa.miR.301a.3p	-0.72	5.70	0.0013	0.017
hsa.miR.181d.5p	-0.72	4.84	0.004	0.029
hsa.miR.181a.5p	-0.70	11.11	0.0004	0.010
hsa.miR.20a.5p	-0.61	9.16	0.0006	0.011
hsa.miR.374b.5p	-0.58	6.65	0.008	0.046
hsa.miR.374a.5p	-0.56	8.63	0.0006	0.011
hsa.miR.452.5p	-0.52	4.99	0.007	0.041
hsa.miR.92a.3p	-0.51	12.93	0.008	0.046
hsa.miR.101.3p	-0.45	12.06	0.004	0.031
hsa.miR.19b.3p	-0.44	8.14	0.003	0.022
hsa.miR.30a.5p	-0.40	13.56	0.002	0.019
hsa.miR.30e.3p	-0.39	9.67	0.0001	0.006
hsa.miR.27b.3p	-0.36	12.86	0.0011	0.016
hsa.miR.454.3p	-0.36	9.18	0.008	0.046
hsa.miR.30e.5p	-0.35	12.08	0.002	0.019
hsa.miR.30a.3p	-0.34	10.66	0.0012	0.017
hsa.miR.203a	-0.31	12.47	0.009	0.046
hsa.miR.26b.5p	-0.29	14.30	0.007	0.043
hsa.miR.26a.5p	-0.25	15.67	0.002	0.019

FC, fold change; CPM, counts per million; FDR, false discovery rate; miR, microRNA

Table S3. Significantly differentially expressed miRNA measured by RT-qPCR between COPD subjects and healthy ex-smokers, N=44

miRNA	COPD SD	Healthy_ES SD	Log2FC	P value	FDR
**Upregulated in COPD**					
**hsa-miR-2110**	1.21	0.71	2.12	0.001	0.016
**hsa-miR-223-3p**	1.47	1.37	2.97	0.001	0.016
**hsa-miR-625-3p†**	0.91	0.76	1.85	0.006	0.041
**hsa-miR-182-5p**	0.70	0.66	1.52	0.006	0.041
**hsa-miR-200b-5p**	0.79	0.72	1.52	0.009	0.047
**Downregulated in COPD**					
**hsa-miR-204-5p**	1.32	1.23	-2.37	0.003	0.037
**hsa-miR-138-5p**	0.90	0.77	-1.66	0.005	0.041
**hsa-miR-338-3p**	1.15	0.78	-1.72	0.009	0.047

Shapiro-Wilk test for normality was performed and showed data were normally distributed. Unpaired Welch's t test was performed and then adjusted using Benjamini Hochberg to generate an FDR value. COPD, Chronic obstructive pulmonary disease, FC: Fold change. FDR, false discovery rate; Healthy_ES, healthy ex-smoker; miRNA, microRNA, SD: standard deviation.†missing data points; COPD, n=18; Healthy-ES, n=12

Table S4. Logistic regression of proportions of miRNA reads in COPD and healthy ex-smokers

Variable	OR (95% CI)*	P Value
Age in years	1.05 (0.9-1.2)	0.5
Smoking pack years	1.03 (0.9-1.1)	0.3
Gender	2.7 (0.02-30)	0.4
Lobe sampled	1.8 (0.03-2.4)	0.2
**miRNA read %**	**1.2 (1.02-1.3)**	**0.02***

*Without unmapped reads included. CI, confidence interval; miRNA, microRNA; OR, Odds ratio

Table S5. ROC analysis for predictive ability of up-regulated miRNA to differentiate between COPD and healthy ex-smokers

miRNA	Log2FC	AUC (95% CI)	Standard Error[a]	Cut-off point	Sensitivity	Specificity	P value
**miR-2110**	2.12	**0.81** (0.68 - 0.93)	0.06	-3.59	**65.4**	**90**	**<0.0001**
**miR-223-3p**	2.97	**0.79** (0.65 – 0.93)	0.07	-3.22	**73.1**	**75**	**0.001**
**miR-182-5p**	1.52	**0.78** (0.64 – 0.92)	0.07	-4.73	**65.4**	**90**	**0.001**
miR-625-3p[Γ]	1.85	0.76 (0.59 – 0.93)	0.09	-8.29	73.1	50	0.02
miR-200b-5p	1.52	0.71 (0.56 – 0.87)	0.08	-2.60	65.4	75	0.02
Combinations of up-regulated EV miRNA							
**miR-2110, miR-223-3p, miR-182-5p**	NA	**0.91** (0.8 - 0.98)	0.05	-12.28	**80.8**	**90**	**<0.0001**
miR-2110, miR-223-3p, miR-182-5p, miR-200b-5p	NA	0.85 (0.73 – 0.97)	0.06	-14.65	76.9	90	<0.0001
miR-2110, miR-223-3p	NA	0.84 (0.72 – 0.96)	0.06	-7.14	80.8	85	<0.0001
miR-2110, miR-182-5p	NA	0.84 (0.73 – 0.96)	0.06	-8.38	61.5	95	<0.0001
miR-223-3p, miR-182-5p	NA	0.83 (0.72 – 0.96)	0.06	-8.00	73.1	90	<0.0001

[Γ] data missing for 13 COPD subjects, N=11. [a.] under the nonparametric assumption. AUC, area under receiver operator characteristic curve; CI, confidence interval; Cut-off point = where sensitivity + specificity – 1 is maximal; miRNA, microRNA; NA, non-applicable

Table S6. Correlations between EV miRNA expression and immune cells proportions within BALF of COPD subjects

**BALF EV miRNA**	**Neutrophils %**	**Eosinophils %**
**Upregulated in COPD**		
miR-2110	**0.47***	0.2
miR-223-3p	0.35	**0.47***
miR-182-5p	**0.46***	0.1
miR-625-3p[Γ]	0.12	0.23
miR-200b-5p	0.33	0.02
**Downregulated in COPD**		
miR-204-5p	**-0.49***	**-0.57****
miR-138-5p	-0.11	-0.22
miR-338-3p	-0.22	**-0.42***

[Γ]missing data for 13 COPD subjects, N=11. Spearman's correlation coefficient. N = 24 *p<0.05, **p<0.005. BAL; bronchoalveolar lavage fluid; EVs, extracellular vesicles; miR, microRNA.

Table S7. Definitions of inflammatory endotypes in COPD using American Thoracic Society Clinical Practice Guidelines for BAL analysis pre-defined cut-offs.

Inflammatory endotype	% Neutrophils in BALF	% Eosinophils in BALF
Eosinophilic	NA	>1%
Neutrophilic	>3%	NA
Mixed granulocytic	>3%	>1%
Paucigranulocytic	≤3%	≤1%

BALF, bronchoalveolar lavage fluid; NA, non-applicable

Table S8. ROC analyses to determine predictive ability of miRNA to differentiate between neutrophilic and non-neutrophilic subtypes in COPD

miRNA	AUC (95% CI)	Standard Error[a]	P value
miR-2110	0.69 (0.5 – 0.9)	0.12	0.2
miR-223-3p	0.67 (0.4 – 0.9)	0.12	0.2
miR-182-5p	0.58 (0.3 – 0.9)	0.14	0.6
miR-625-3p[Γ]	0.34 (0.09 – 0.6)	0.13	0.2
miR-200b-5p	0.52 (0.2 – 0.8)	0.14	0.9
miR-204-5p	0.60 (0.4 – 0.8)	0.12	0.4
miR-138-3p	0.4 (0.2 – 0.6)	0.12	0.4
miR-338-3p	0.44 (0.2 – 0.7)	0.12	0.6

[a.] Standard error under the nonparametric assumption
[Γ] data missing for 13 subjects

4 Supplementary Figures

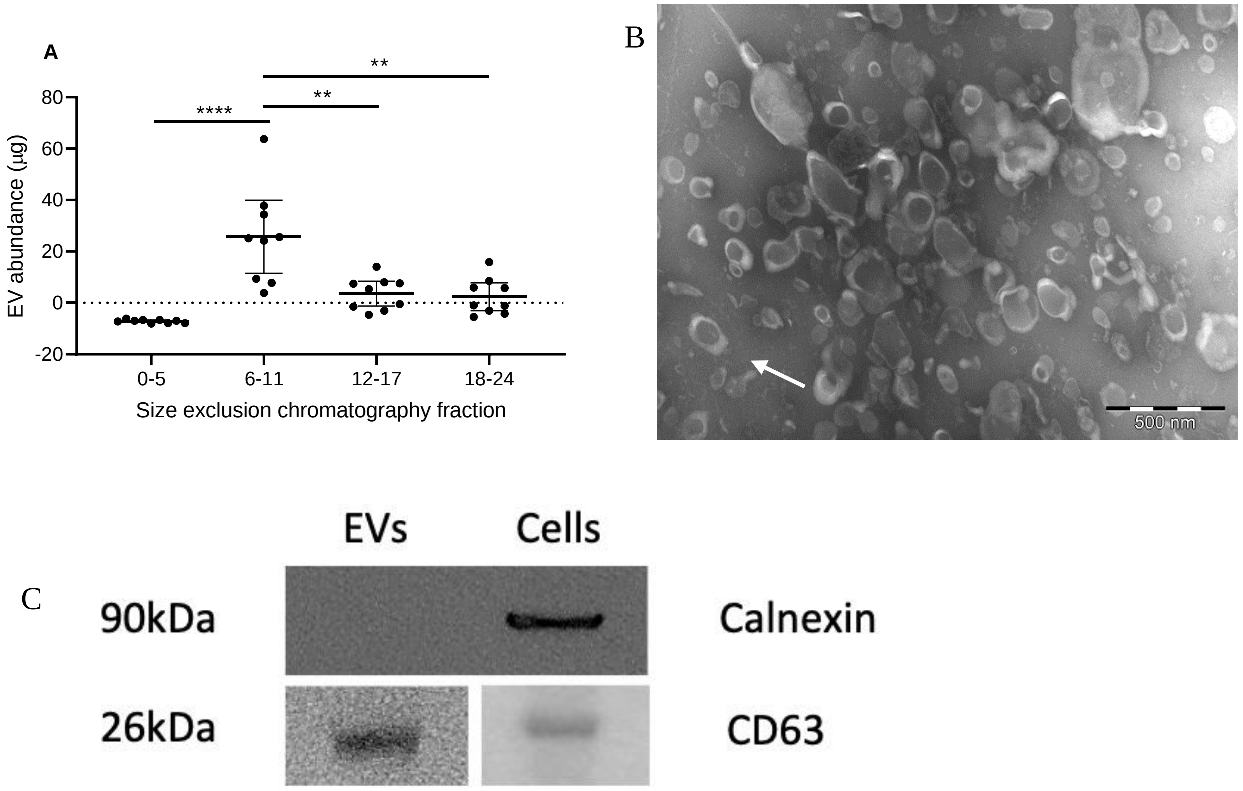

Figure S1. (A) EV abundance according to presence of CD9 in combined SEC fractions, with fractions 6-11 corresponding to EV containing fraction. (B) Whole mounted lung-derived EVs isolated from fractions 6-11 using SEC viewed by transmission electron microscopy. White arrow points to a characteristic EV with cup-shaped morphology and size between 30 – 150 nm. Scale bar shown in bottom right-hand corner. BALF, bronchoalveolar lavage fluid; EV, extracellular vesicles; SEC, Size exclusion chromatography. (C) Western blot analysis of isolated BALF EVs. 30ug of protein was used for Western blot analysis. Images captured from different WB for CD63 due to issues with running gel non-reducing conditions.

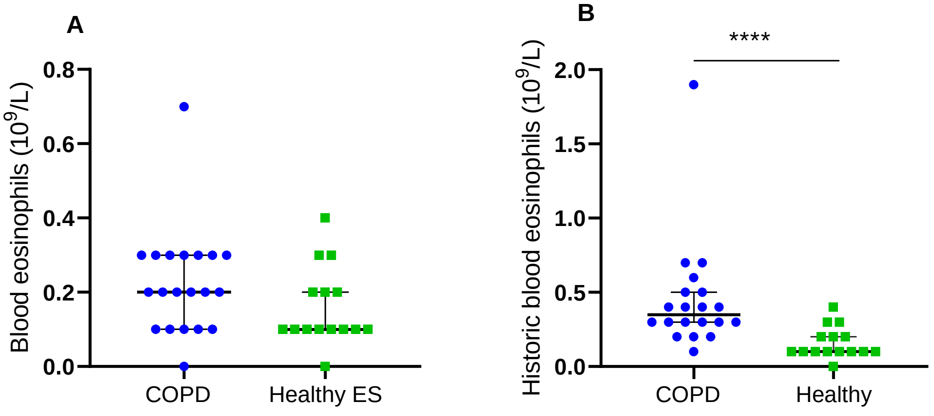

Figure S2. Enrolment (A) and highest-ever recorded (B) blood eosinophil count for COPD subjects compared with healthy ex-smokers. Median and IQR presented as skewed data. ****p<0.0001 using Mann Whitney test

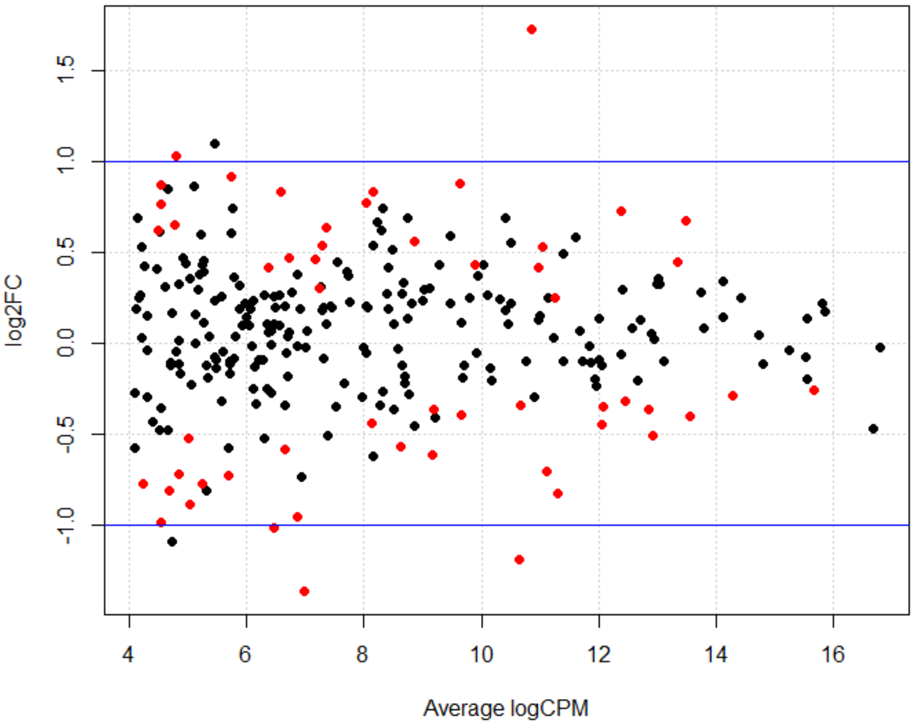

Figure S3. MA plot showing differentially expressed miRNA between COPD subjects and healthy ex-smokers based on RNA sequencing analysis. N=31. Red dots represent miRNA with an FDR <0.05. Blue lines represent a twofold change in expression. CPM, counts per million; FC, fold change.

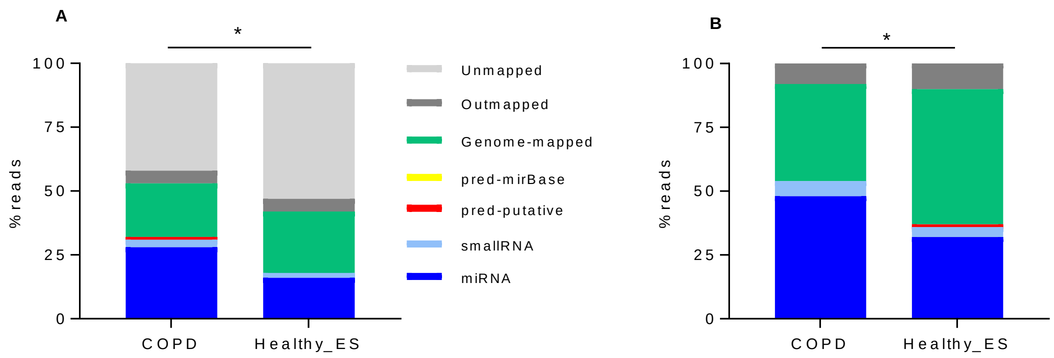

Figure S4. Proportions of different types of reads in COPD and healthy ex-smoker samples
A includes unmapped reads and B excludes unmapped reads. N=31; COPD, n=17. Chi-squared test performed on proportion of miRNA present in two groups, *p<0.05. COPD, Chronic obstructive pulmonary disease; Health-ES, healthy ex-smoker; miRNA, microRNA; Pred, predicted

frontiers
